# Predictors of patient safety competency among emergency nurses in Iran: a cross-sectional correlational study

**DOI:** 10.1186/s12913-022-07962-y

**Published:** 2022-04-25

**Authors:** Aghil Habibi Soola, Mehdi Ajri-Khameslou, Alireza Mirzaei, Zahra Bahari

**Affiliations:** 1 Department of Nursing, School of Nursing and Midwifery, Ardabil University of Medical Sciences, Ardabil, Iran; 2 Department of Critical Care Nursing, School of Nursing and Midwifery, Ardabil University of Medical Sciences, Ardabil, Iran; 3 Department of Emergency nursing, School of Nursing and Midwifery, Ardabil University of Medical Sciences, Ardabil, Iran

**Keywords:** Patient safety competency, Teamwork, Team structure, Leadership, Psychological safety Emergency nurses

## Abstract

**Aims:**

This study aimed to assess predictors of patient safety competency among emergency nurses.

**Background:**

The role of emergency nurses is to provide high-quality health care to patients and ensure their safety. The patient safety competency includes the absence of unnecessary or potential harm when providing health care to patients. In providing health care, effective teamwork can affect patient safety and outcomes. Psychological safety is essential to effective teamwork. Psychological safety allows health care workers to accept the interpersonal risks needed to perform effective teamwork and maintain patient safety.

**Methods:**

This study was cross-sectional correlational research. Using convenience sampling methods, 254 emergency department nurses from five educational hospitals were enrolled in the study. Patient Safety in Nursing Education Questionnaire was used to measure the patient safety competency, the teamwork questionnaire to examine the teamwork, and Edmondson psychological safety questionnaire was used to measure psychological safety. Descriptive statistics, t-test, one-way analysis of variance (ANOVA), Pearson’s r correlation coefficient, and multivariate stepwise linear regression analysis were applied using SPSS 14.0.

**Results:**

Participants’ mean patient safety competency score was 2.97 (1-4). Between 18 independent variables evaluated in the multiple regression analysis, seven had a significant effect on the patient safety competency of emergency nurses (R^2^: 0.39, *p* < .001).

**Conclusions:**

The patient safety competency of emergency department nurses was primarily related to the structure and leadership of the team and secondary to psychological safety and experience in patient safety activity. The results demonstrated that policymakers and hospital managers should improve and enhance team structure and leadership via supervision and cooperation with the nursing staff. The development of training programs in patient safety activities, improvement, and increase of psychological safety at the levels of the nursing units is essential to increase patient safety competencies in the emergency nursing program.

## Introduction

In recent years, patient safety has been considered an important issue in the health care system [1]. Medical error and adverse events occur with considerable frequency in hospitals. Also, the unpredictability and complex nature of emergency departments (EDs), the critical condition of most patients, lack of teamwork, and large volumes of work due to the inadequacy of the patient and nurse ratios convert the emergency department to a high-risk area for patient adverse events [2]. The prevalence of adverse events in ED is higher and varied from 4 to 68% [3]. According to a study by Kakemam et al., the prevalence of adverse events in Iran is between 7 and 40% [4]. Lower adverse events and better patient care safety are usually the outcomes of perceived teamwork [5].

Teamwork is very important due to the critical work environment, the large number of staff, and the complexity of work in the ED [5, 6], and was suggested as a solution to improve the quality of care and safety of patients in the emergency department [7]. Effective teamwork improves team efficiencies and patient safety, leads to a healthier and happier workplace, and reduces job burnout among healthcare professionals [8, 9]. According to a study by Ajri-Khameslou et al., emergency nurses believe that teamwork may help increase the level of efficiency and safety of patient care in the emergency department [10]. Considering the increase in demand, emergency departments need to improve efficiency while providing safe and effective care. Providing efficient and quality health care is affected by interactions among physical structure, processes, and emergency department results [11]. One of the key components to improve teamwork is psychological safety [12]. A study by O’Donovan found that psychological safety is a key determinant of high-quality communication that improves teamwork and, therefore, plays an important role in healthcare teams [12].

Psychological safety is defined as the conditions under which team members feel safe to take risks, discover new ideas, and challenge the status quo [12, 13]. In fact, the team is ensured that group members are not blamed for report errors. High psychological safety represents a safe environment, interpersonal trust, mutual respect and talks about safety. Psychological safety reduces the fear of rejection and supports active participation, creating an environment that which team members probably recognize errors and resolve disadvantages and shortcomings (13, 14). The psychological safety of team members strengthens creativity and active participation in teamwork. Also, providing psychological safety to nurses increases patient safety and quality of care and lower levels of psychological safety led to increased psychological stress, absenteeism, endangering patient safety, and reducing job satisfaction in Nurses [13, 14]. The results of the study Green et al. showed that the psychological safety of health workers in the care environment influences teamwork, engagement in quality improvement work, learning from failures, reporting adverse events, and patient safety competency [14].

Patient safety competency refers to the attitudes, skills, and knowledge that health care workers must have to protect patients from unnecessary risks and hazards and is crucial in nurses because they are responsible for the 24-h care of patients [2]. According to Tella et al., the main content of the patient’s safety in contemporary nursing education includes the competency to prevent patient safety accidents (attitude), the creation of patient safety competency (knowledge), and the competency to act after the error (skill) [15]. Results of a study showed that the weakness in the emergency department patient safety is due to the lack of optimal reports of adverse events, lack of teamwork in the units, and the transfer of patients [16].

Research about predictors of patient safety competency among emergency nurses is limited [2]. as most of the literature is related to patient safety competency in nursing students and recent graduates [1, 8, 15]. In the study by Han et al., situation monitoring, psychological safety, and the reporting of patient safety adverse events were the main factors predicting patient safety competency. Also, they suggested that these aspects need to be improved [2].

Findings from Hwang et al. demonstrated that teamwork received the lowest ranking of the six dimensions of patient safety competency [8]. The results of this study highlighted the need to improve teamwork among nurses in different hospital wards. With the increasing complexity of health care systems, effective teamwork seems essential to ensure patient safety and quality of care [8]. Therefore, improving the safety competency of nurses can help improve patient safety [8]. Teamwork does not always lead to good results. Sometimes caused conflicts among team members or different teams, dysfunctional relationships among employees, disregard, and disrespect for other team members. As a result, it’s lead to negligence in patients’ care [5]. Health care teams need psychological safety for effective teamwork and safe patient care [12].

Due to the importance of patient safety competency and emphasis on teamwork, as well as the importance of psychological safety and attention to it in emergency nurses, and the limited of study in this context in the emergency departments of Iranian hospitals, this study aimed to assess factors that predict the patient safety competency of emergency nurses.

## Methods

### Study design

The study was designed as a questionnaire-based cross-sectional correlational study.

### Study setting and participants

The setting for this study was five emergency departments of Ardabil Teaching Hospitals, northwest of Iran. Among these five hospitals, two hospitals provide generalized services for their patients, and three hospitals provide specialized services. The total number of inpatient bed count in the emergency department in the studied hospitals was 220 beds. The Inpatient Bed Occupancy Rate before the health system reform plan was 75% on average. This rate reaches 95% and sometimes 100% after the start of the health system reform plan. In emergency departments, the division of nurses’ work is done in writing and as a case method. The study population included all nurses working in the emergency departments of generalized and specialized hospitals of city of Ardabil. Ardabil city has five educational hospitals, including 3 generalized centers and 2 specialized centers. The total number of nurses working in the emergency departments of the studied hospitals was 320 nurses. The inclusion criterion was having at least 12 months of experience in the emergency department, a bachelor’s or higher degree in nursing, and a willingness to participate in the study. New graduate nurses with less than one year of experience were excluded from the study. Paper-based questionnaires were distributed by the researchers among 290 nurses eligible to participate in the study. Finally, 254 emergency nurses were enrolled in the study. The response rate was 87% (254/290).

### Study tools

Data collection tools included the demographic and professional information form of participants and patient safety competency questionnaire, teamwork, and psychological safety.

### Demographic information form

Questionnaire of demographic and professional information includes the items of age, gender, level of education, type of hospital, type of emergency department, number of night shifts per month, the total number of years of work experience, the total number of years of emergency work experience, job satisfaction, training on the patient safety (yes/no), experienced in patient safety activities and reporting of patient safety adverse events (adverse event reported by self and those reported by others). Job satisfaction was measured using a question (how satisfied are you about your job?) on the 5-point Likert scale (1 = high dissatisfaction, 5 = high satisfaction) were measured.

### Patient safety competency

One of the valid tools in measuring safety competency is the Patient Safety in Nursing Education Questionnaire [15]. This scale evaluates the understanding of nurses from the patient’s safety based on three categories: Academic conditions (19 items), patient safety in clinical conditions (17 items), and patient safety competency (14 items). To measure the patient safety competency, the third part of the safety questionnaire was used in nursing education. Langari et al. examined the validity and reliability of the third part of the patient safety questionnaire in nursing education. And in the Exploratory analysis factor, they showed that three factors constitute the patient’s safety competence, which was: creating patient safety competency (knowledge), performance competency after the error (skill), and competency to prevent patient safety incidents (attitude) [17]. They also reported Cronbach’s alpha coefficient of this questionnaire as 0.89 [17]. After obtaining permission from the original designer, the English version of this questionnaire was translated into Persian by two independent translators. To determine the index and content validity ratio, the questionnaire was given to 12 faculty members of Ardabil University of Medical Sciences. The content validity index (CVI) was separately evaluated by experts through three criteria of simplicity, appropriateness and certainty based on a four-part spectrum (for example, in terms of simplicity, quite simple, somewhat complex and complex) for each question and the corresponding ratings were given. Finally, the content validity index was 0.91. Cronbach’s alpha coefficient of 0.90 also showed the reliability of the questionnaire. This questionnaire consists of three subgroups of knowledge (4 items), skill (4 items), and attitude (6 items) and follows the 4-point Likert scale (completely disagree = 1 to completely agree = 4).

### Teamwork

Teamwork questionnaire (STEPPS (T-TAQ) was developed by Baker et al. [18] to identify people’s attitudes about teamwork. The tool has 30 questions in five subscales: The team structure, leadership, mutual support, Situation monitoring, and communication. Each subscale contains six items measured on a 5-point Likert scale (1 = strongly disagree to 5 = strongly agree). Questions 20, 21, 24 in the mutual support subscale and question 30 in the communication subscale are scored in reverse [18]. This tool has been translated into Persian, and its reliability has been confirmed and reported at 0.80 using Cronbach’s alpha method [19]. Its Cronbach’s alpha coefficient was 0.93 in the present study.

### Psychological safety

The psychological safety scale was designed by Edmondson [20] to assess psychological safety in teamwork. This questionnaire consists of 7 items, seven-point Likert scale questions (completely disagree = 1, completely agree = 7). Questions 1, 3, and 5 are scored in reverse, and higher scores on the psychological safety Questionnaire indicate the respondent perceives a higher level of team psychological safety [20]. The Persian version of the psychological safety questionnaire was examined by Shams et al. [21]. Their results indicated that the Cronbach’s Alpha for the total scale was 0.81. In this research, Cronbach’s Alpha coefficient study obtained 0.57. This did not meet the criteria of Cronbach’s alpha above 0.7. No items need to be deleted in order to be able to increase Cronbach’s alpha. The small Cronbach’s alpha might be the result of a small-scale size. Since this scale is already validated by Edmondson [20], it was decided to still use the scale despite the low Cronbach’s alpha.

### Data collection and ethical considerations

The study was conducted in accordance with the Declaration of Helsinki Ethical Principles and Good Clinical Practices was approved at each site by Ardabil University of Medical Sciences with the ethical code (IR. ARUMS REC.1399.458). The researchers referred to Imam Khomeini, Imam Reza, Fatemi, Bouali, and Alavi hospitals in Ardabil and were introduced to emergency nurses by the nursing offices of the mentioned centers. Informed written consent was obtained from all participants before the start of this study. By completing the consent form, participants were informed about the purpose and method of the study. Participants were also informed that the researchers are committed to answering their questions and that their information was kept confidential. In addition, participants were aware that their participation in the study was voluntary and that they could leave the study at any time. then, the paper version of the questionnaire was distributed by the researchers to the emergency nurses who had given their written consent to attend the study. Information was collected from February 1 to 1 April 2021.

### Data analysis

Data were analyzed using SPSSV14 software. The level of teamwork, psychological safety, and patient safety competency in emergency nurses was analyzed using descriptive statistics. The interactions of teamwork, psychological safety, and patient safety competency of emergency nurses were analyzed using Pearson correlation. The relationship between patient safety competency and demographic characteristics was investigated using an independent t-Test, ANOVA, and Pearson correlation. The predictive factors of patient safety competency from emergency nurses using multiple stepwise regression were investigated.

## Results

Descriptive results showed that most participants (56.7%) were female and married (66.9%). The majority of participants (83.1%) had a bachelor’s degree. Also, 51.6% of emergency nurses were working in specialized hospitals. Half of the participants had less than or equal to 60 months of work experience and the type of employment of the majority (61.1%) was employed. The following demographic characteristics of the respondents are shown in (Table 1).

**Table 1:** General characteristics of Emergency nurses (*N* = 254)

Characteristics	Categories	Mean	SD
**Age (years)**		31.70	6.30
**Job satisfaction**		3.35	1.27
		n	%
**gender**	Male	110	43.3
	Female	144	56.7
**Marital status**	Unmarried	84	33.1
	Married	170	66.9
**Education level**	Bachelor’s degree	211	83.1
	Master’s degree and PhD	43	16.9
**Type of employment**	Commitment	56	22.0
	Contractual	43	16.9
	Employed	155	61.1
**Type of hospital**	General Hospital	123	48.4
	specialized hospital	131	51.6
**Type of emergency department**	Trauma Emergency	88	34.6
	Internal emergency	139	54.7
	Maternal and child emergency	27	10.6
**Number of night shifts per month**	≤6	91	35.8
	≥7	163	64.2
**Total work experience (months)**	≤60	127	50.0
	61 - 120	73	28.7
	≥121	54	21.3
**Total work experience in emergency department (months)**	≤60	187	73.6
	61 - 120	49	19.3
	≥121	18	7.1
**Training in patient safety**	Yes	187	73.6
	No	67	26.4
**Experience in patient safety activity**	Yes	154	60.6
	No	100	39.4
**Reported adverse event by self**	Yes	118	46.5
	No	136	53.5
**Reported adverse event by others**	Yes	157	61.8
	No	97	38.2

### Descriptive statistics and relationship between patient safety competency and teamwork and psychological safety

Table 2 shows the descriptive statistics and the relationship among patient safety competency, teamwork, and its subgroups, and psychological safety of emergency nurses. The mean and standard deviation (SD) for patient safety competency, teamwork, and psychological safety were 2.97 (0.54), 3.60 (0.56), and 27.04 (4.67), respectively. Patient safety competency had a positive correlation with teamwork (*r* = 0.4873, *P* < .001), and psychological safety (*r* = 0.167, *P* < 0.001). Furthermore, the patient safety competency had a positive correlation with leadership, communication, mutual support, situation monitoring, and team structure (*P* < 0.001).

**Table 2:** Descriptive statistics and correlations among the study variables (*N* = 254)

Variable			Patient safety competency
	Mean ± SD	Min/Max	r (p)
Teamwork			
Leadership	3.81 ± 0.78	1-5	0.498 (< 0.001)
Communication	3.51 ± 0.62	1-5	0.409(< 0.001)
Mutual support	3.26 ± 0.41	1-5	0.349 (< 0.001)
Situation monitoring	3.75 ± 0.75	1-5	0.447 (< 0.001)
Team structure	3.65 ± 0.77	1-5	0.571 (< 0.001)
Total	3.60 ± 0.56	1-5	0.556 (< 0.001)
Psychological safety	27.04 ± 4.67	9-39	0.167 (< 0.001)
Patient safety competency			
Knowledge	2.94 ± 0.65	1-4	
Skills	2.96 ± 0.58	1-4	
Attitudes	3.00 ± 0.53	1-4	
total	2.97 ± 0.54	1-4	1

### Relationship between patient safety competency and general characteristics of emergency nurses

Table 3 shows the relationship between patient safety competency and general characteristics of emergency nurses. Patient safety competency with age (*r* = 0.170, *p* = 0.007), job satisfaction (F = 3.475, *p* = 0.009), level of education (t = 3.290, *p* = 0.001), type of employment (F = 2.678, *p* = 0.048), type of emergency (t = 3.602, *p* = 0.029), training in patient safety (t = 3.256, *p* = 0.001), experience in patient safety activity (t = 4.635, *p* < .001) and reported adverse event by others (t = 3.247, *p* = 0.001), had a significantly positive correlation. Also, patient safety competency was significantly negative correlation with the type of hospital (t = −5.224, *p* < .001).

**Table 3:** Relationship between nurses’ general characteristics and patient safety competency. (*N* = 254)

Characteristics	Categories	Mean	SD	Test statistics	*p* value
Age (years)^a^		2.97	0.54	0.170	0.007
Job satisfaction^c^	very dissatisfied	2.88	0.64	3.475	0.009
	A little dissatisfied	2.91	0.37		
	No idea	2.77	0.63		
	A little satisfied	3.12	0.46		
	very satisfied	2.97	0.62		
Gender^b^	male	2.93	0.55	−0.872	0.384
	female	2.99	0.52		
Marital status^b^	Unmarried	2.95	0.57	−0.354	0.723
	Married	2.98	0.52		
Education level^b^	Bachelor’s degree	3.02	0.51	3.290	0.001
	Master’s and PhD	2.72	0.61		
Type of employment^c^	Commitment	3.04	0.48	1.776	0.173
	Contractual	3.06	0.49		
	Employed	2.92	0.57		
Type of hospital^b^	generalized hospital	2.79	0.50	−5.224	0.000
	specialized hospital	3.13	0.52		
Type of emergency department^c^	Trauma Emergency	3.09	0.52	3.602	0.029
	Internal emergency	2.91	0.53		
	Maternal and child	2.86	0.62		
Number of night shifts per month^b^	≤6	3.03	0.56	1.394	0.164
	≥7	2.93	.52		
Total work experience (months)^c^	≤60	2.89	0.58	2.899	0.057
	61 - 120	3.01	0.47		
	≥121	3.09	0.51		
Total work experience in emergency department (months)^c^	≤60	2.95	0.54	0.300	0.741
	61 - 120	3.01	0.48		
	≥121	3.02	0.63		
Training in patient safety^b^	Yes	3.03	0.56	3.256	0.001
	No	2.79	0.42		
Experience in patient safety activity^b^	Yes	3.09	0.56	4.635	0.000
	No	2.78	0.45		
Reported adverse event by self^b^	Yes	3.04	0.55	1.917	0.056
	No	2.91	0.52		
Reported adverse event by others^b^	Yes	3.05	0.54	3.247	0.001
	No	2.83	0.50		

^a^Pearson r correlation

^b^t test for independent group

^c^Analysis of variance

### Factors predicting patient safety competency

A multiple stepwise regression analysis was performed using patient safety competency as the dependent variable and general characteristics, teamwork, and psychological safety as independent variables. The Durbin–Watson test statistic was 1.51, thereby falling within 1.5–2.5; as such, there was no linear relation in the data. The variance inflation factor ranged from 1.07 to 3.43, whereas tolerance ranged from 0.29 to 0.98 with no multicollinearity among independent variables. Subscales of teamwork (team structure, leadership, communication, mutual support, situation monitoring), psychological safety, age, gender, marital status, experience ED, job satisfaction, education level, type of employment, work position, training in patient safety, experience in patient safety activity, reported adverse event by self and reported adverse event by others in sequence. Among these 18 variables, seven accounted for 40.9% of the variance in the final model (F = 24.291, *p* < .001). Thus, the significant predictors of patient safety competency were, team structure (β = 0.374, *p* < .001), leadership (β = 0. 188, *p* = 0.012), age (β = 0.177, *p* = 0.003), type of employment (β = −0.135, *p* = 0.022), experience in patient safety activity (β = 0.128, *p* = 0.014), psychological safety (β = 0.125, *p* = 0.013) and gender (β = 0.105, *p* = 0.037). Significant predictor variables of patient safety competency are shown in Table 4.

**Table 4:** Stepwise multiple regression related to patient safety competency (*N* = 254)

Variable	*B*	SE	Beta	t	*p*	ΔR^2^
**(Constant)**	0.831	0.296		2.809	0.005	
**Team structure**	0.262	0.053	0.374	4.914	*p* < 0.001	0.326
**Leadership**	0.130	0.052	0.188	2.519	0.012	0.361
**Experience in patient safety activity**	0.142	0.058	0.128	2.472	0.014	0.345
**Psychological safety**	0.015	0.006	0.125	2.494	0.013	0.373
**age**	0.015	0.005	0.177	2.966	0.003	0.386
**Type of employment**	−0.061	0.026	−0.135	−2.307	0.022	0.398
**gender**	0.115	0.055	0.105	2.102	0.037	0.409

AdjustedR^2^ = 0.39, F = 24.291, *p* < 0.001

## Discussion

In the emergency department, patient safety relies on teamwork and coordination between clinical groups, as emergency nurses provide front-line care. Therefore, patient safety competency is crucial to ensure quality safe care. In our opinion, this is the first study that assesses the predictors of patient safety competency among Iranian emergency nurses using valid tools and, therefore, adds new knowledge in this field.

The patient safety competency level of emergency nurses in our study was 2.97, which was slightly lower (3.53) than the findings of Langari et al., [17]. Also, this result was slightly higher (3.76) than the findings of the study of Han & Roh [2]. These findings can be explained by cultural differences in patient safety education worldwide. The results of Han’s study showed that emergency nurses focus mainly on patient-related emergency activities and have a relatively small understanding of patient safety. Therefore, they need patient safety management activities, training, standardization, and measures to cope with problems [2]. Langari et al., in their study, showed that European countries (Britain and Finland) have started patient safety and education activities for many years. Therefore, these results may be related to the initial integration of patient safety in the nursing curricula of these countries [17]. These measures have led to improved nurses’ safety competency. Therefore, in the Iranian health system, it is necessary to integrate patient safety competence into nursing curricula. Also, nursing educators should strengthen the patient’s patient safety competency of emergency nurses by developing structured training programs. Nursing managers should adopt reliable tools to regularly assess patient safety competency that can guide safety policy development and training interventions. There is also a need to develop a specialized curriculum to strengthen evidence-based nursing performance and competencies for continuous quality improvement of nurses. Nurses should also learn to encourage patients to participate in patient safety. In addition, it is essential for hospital administrators to make patient safety education as common as possible.

The teamwork score among emergency nurses obtained in this study, was slightly lower than the study of Han & Roh [2] using similar tools. In another study in Iran, the score of teamwork among emergency nurses was slightly lower than our study [5]. Nurses’ perception of teamwork is related to patient safety [22]. Teamwork leads to more employee job satisfaction, increased patient safety, improved quality of care, and greater patient satisfaction [9]. Effective teamwork in healthy environments also helps care for high-quality patients [6]. Besides, safe and quality patient care can also result from effective teamwork [9]. Teamwork is the cornerstone of quality care and patient safety [7]. The results of our study show the need to improve the teamwork of emergency nurses. Emergency nurses can promote teamwork by facilitating communication, resolving disputes among team members, clarifying roles and responsibilities, and encouraging other team members. Nursing managers can promote teamwork by facilitating communication, managing conflict between team members, expanding the nursing team into other specialized areas, clarifying roles and responsibilities, and encouraging other team members. Nurses can also learn effective communication skills. They can also be the first to promote teamwork between themselves and other colleagues.

The psychological safety of emergency nurses was lower than the results of a similar study in South Korea [2]. Psychological safety plays a special role in high-risk work areas such as health care [13]. When health care teams are psychologically safe, they are likely to improve the quality and teamwork initiatives [14]. Psychological safety supports patient safety by contributing to quality improvement and encouraging employees to talk about mistakes [23]. Promoting psychological safety among health care workers increases patient safety and improves the quality of health care.

The results of stepwise regression revealed that team structure (β = 0.374, *p* < 0.001), leadership (β = 0.188, *p* = 0.012) and psychological safety (β = 0.125, 0.013). = p) were the organizational factors affecting patient safety competency. The emergency department is a potentially unique environment for teamwork and communication [10]. The team structure is an integral part of the teamwork process. A properly structured patient care team is an enabler for and the result of effective communication, leadership, situation monitoring, and mutual support. Proper team structure can promote teamwork by including a clear leader, involving the patient, and ensuring that all team members commit to their roles in effective teamwork [6].

Organized and effective teamwork in the emergency department has extensive implications on patient safety, quality of care, employee and patient satisfaction. A team with a proper teamwork structure can predict the requirements of other team members. It is dynamically compatible with a changing environment including changing team members’ behavior and a common understanding of what should happen [9, 24]. The nurses who consider their managers as strong leaders can empower their working environment, which, in turn, leads to their use of professional practice behaviors. These practical professional behaviors, such as effective communication, cooperation, and mutual understanding with clinical leadership’s main characteristics, are compatible [25]. The findings obtained by Boamah [24], showed that nurses often use clinical leader behaviors in their practice, which leads to the improvement of the patient’s adverse events. To ensure the patient’s safety, strong nursing leadership for implementation and continuity of effective management methods for training and continuous support for the environment is required to provide high-quality patient care. In our study, leadership has the largest teamwork score and is one of the predictors of patient safety competency. There are many factors, including leadership, communication, monitoring, and supporting behavior which helps create ideal teamwork in the emergency department [10]. The findings of the study of Parr et al., show that leaders improve patients’ satisfaction with patient services and safety [26].

Based on the findings obtained by Kakemam et al. [22], nurses with stronger team leadership tend to report adverse events. Moreover, the result obtained by Kakemam showed that reducing medical errors can be achieved by effective leadership. Effective leadership can develop and strengthen the patient’s safety and innovation culture in the health care environment. Accordingly, leaders are recommended to use educational strategies for reducing adverse events [22]. The results obtained by Labrague et al., indicate the importance of the advancement of nurses’ management in strengthening nurses’ safety measures reducing the results of the patient and promoting the quality of nursing care [27]. The results show that nursing leadership has a significant indirect effect on patient safety outcomes. From a person-centered perspective, the care environment requires workplace empowerment and effective relationships between leaders and nurses [28]. Effective teamwork and leadership are recognized as important factors in many adverse events. Thus, a greater understanding of team dynamism and effectiveness and helping improve group training can lead to patient safety development and strengthen individual and team competencies in non-technical aspects of care, such as prioritization, leadership, and decision-making.

Having safe teams psychologically can improve learning, creativity, and performance in organizations. In the framework of healthcare, psychological safety supports patient safety by creating the ability to promote quality and encourage employees to talk about medical errors [23]. Psychological safety has been recently a critical factor in understanding phenomena such as teamwork, team learning, and organizational learning [23, 29]. Effective teamwork depends on the psychological safety of the team members, which are defined as their ability to trust each other and feel sufficient safety in the team to accept the fault, ask questions, and present new data. Higher psychological safety leads to better reports of adverse events [2]. Adopt strategies for improving the psychological safety of emergency nurses are required. When the employees are assured that the organization considers their welfare as the priority, they feel psychological safety and this makes improving the patient’s safety in everyday clinical performance [30]. Providing psychological safety to emergency nurses increases patient safety and the quality of care. Therefore, nursing managers should always strive to develop effective methods to improve the psychological safety of their employees.

Our findings indicated that high age (β = 0.177, *p* = 0.003) was the predictor of the patient safety competency of emergency nurses, such that the safety competency increases with rising age. This finding was consistent with the results obtained by Alquwez and Chen [31, 32]. This finding is probably due to the more professional and responsible behavior of the nurses with increasing age.

Experience in patient safety activities (β = 0.128, *p* = 0.014) was another factor that affected the patient safety competency of emergency nurses. Nurses play a key role in coordinating patient safety activities because they are in close contact with patients and are involved in health care teams’ decisions about patient safety [33]. Having previous information and activities about patient safety can improve nurses’ level of safety knowledge and affect their performance in patient safety [34, 35]. Therefore, nurses the more experience in patient safety activities are in the emergency department, the more committed they will be to patient safety and compliance, and the more competent they will be in terms of patient safety.

Gender (β = 0.105, *p* = 0.037) was also one of the work-related factors predictors of the safety competency of emergency nurses. Hence, the mean score in women was significantly higher than men. These findings were consistent with results obtained by Chen and Jabarkhil [32, 36] and were not consistent with those obtained by Alquwez [31]. One reason for such a difference in outcome can be attributed to the participation of male and female nurses. Nursing is a majority female-dominated profession. In this study, 43.3% of the nurses were male. However, women are generally more sensitive than men to safety, quality of patient care, and the use of safety principles. These results suggest that women pay more attention to patient safety than men.

Emergency nurses with non-permanent employment status (β = −0.135, *p* = 0.022) perceive patient safety competency more than those with permanent employment, which was not consistent with the study by Chegini et al. [37]. It Can be concluded that permanent employees are in the ultimate employment status, and non-permanent employees are more efforts to improve and upgrade their employment status. Therefore, it is necessary to provide in-service patient safety training for this group of employees.

### Limitation

In our opinion, this is the first study that evaluates the predictors of patient safety competency of Iranian emergency nurses. However, there are some limitations to our research that may affect the results and should be addressed when interpreting our findings. First, the discussion of safety in a health institution dos not complete without considering factors such as workplace violence, accreditation status, workload, and disease severity in patients, which may affect nurses’ patient safety competency scores. Therefore, further studies are needed to assess other factors affecting patient safety competency. Second, our sample consisted only of nurses working in the emergency departments of Ardabil teaching hospitals, Iran. Hence, our results should be generalized with caution. Third, as we measured all variables using our reports, our results may include responsive response bias. Finally, the current study was performed using a cross-sectional design that makes it impossible to resolve causal relationships between variables.

## Conclusion

The work environment in emergency care is complex and unpredictable and may affect patient safety. The finding of this study indicated that the total score of emergency nurses’ patient safety competencies was 2.97. Improving and upgrading team structure and leadership, having experience in patient safety activities, and improving and enhancing psychological safety at the nursing unit levels are essential to increase patient safety competencies in emergency nurses. It is recommended that patient safety training be provided to staff during service and put patient safety at the forefront of all nursing activities and practices. Further studies are needed to assess patient safety competency in emergency nurses.

## Data Availability

The datasets used and/or analysed during the current study available from the corresponding author on reasonable request.
