# Correction: Predictors of patient safety competency among emergency nurses in Iran: a cross-sectional correlational study

**DOI:** 10.1186/s12913-022-08016-z

**Published:** 2022-05-16

**Authors:** ibi Soola, Mehdi Ajri-Khameslou, Alireza Mirzaei, Zahra Bahari

**Affiliations:** 1 Department of Nursing, School of Nursing and Midwifery, Ardabil University of Medical Sciences, Ardabil, Iran; 2 Department of Critical Care Nursing, School of Nursing and Midwifery, Ardabil University of Medical Sciences, Ardabil, Iran; 3 Students research committee, Department of Emergency nursing , School of Nursing and Midwifery, Ardabil university of medical sciences, Ardabil, Iran

**Correction to: BMC Health Services Research 22, 547 (2022)**

10.1186/s12913-022-07962-y

Following publication of the original article [1], the authors identified an error in affiliation 3.

The incorrect affiliation 3 is: Department of Emergency nursing, School of Nursing and Midwifery, Ardabil University of Medical Sciences, Ardabil, Iran.

The correct affiliation 3 is: Students research committee, Department of Emergency nursing, School of Nursing and Midwifery, Ardabil university of medical sciences, Ardabil, Iran.

Affiliation 3 is corrected in the affiliation list of this Correction article and the original article [1] has been updated.
